# 3D Bioprinted Osteogenic Tissue Models for In Vitro Drug Screening

**DOI:** 10.3390/molecules25153442

**Published:** 2020-07-29

**Authors:** Erick Breathwaite, Jessica Weaver, Justin Odanga, Myra dela Pena-Ponce, Jung Bok Lee

**Affiliations:** Institute of Regenerative Medicine, LifeNet Health, 1864 Concert Drive, Virginia Beach, VA 23453, USA; erick_breathwaite@lifenethealth.org (E.B.); jessica_weaver@lifenethealth.org (J.W.); justin_odanga@lifenethealth.org (J.O.); myra_delapenaponce@lifenethealth.org (M.d.P.-P.)

**Keywords:** scaffold-free, 3D bioprinting, BM-MSC, bone, drug screening

## Abstract

Metabolic bone disease affects hundreds of millions of people worldwide, and as a result, in vitro models of bone tissue have become essential tools to help analyze bone pathogenesis, develop drug screening, and test potential therapeutic strategies. Drugs that either promote or impair bone formation are in high demand for the treatment of metabolic bone diseases. These drugs work by targeting numerous signaling pathways responsible for regulating osteogenesis such as Hedgehog, Wnt/β-catenin, and PI3K-AKT. In this study, differentiated bone marrow-derived mesenchymal stem cell (BM-MSC) scaffold-free 3D bioprinted constructs and 2D monolayer cultures were utilized to screen four drugs predicted to either promote (Icariin and Purmorphamine) or impair osteogenesis (PD98059 and U0126). Osteogenic differentiation capacity was analyzed over a four week culture period by evaluating mineralization, alkaline phosphatase (ALP) activity, and osteogenesis related gene expression. Responses to drug treatment were observed in both 3D differentiated constructs and 2D monolayer cultures. After four weeks in culture, 3D differentiated constructs and 2D monolayer cultures treated with Icariin or Purmorphamine showed increased mineralization, ALP activity, and the gene expression of bone formation markers (*BGLAP*, *SSP1*, and *COL1A1*), signaling molecules (*MAPK1*, *WNT1*, and *AKT1*), and transcription factors (*RUNX2* and *GLI1*) that regulate osteogenic differentiation relative to untreated. 3D differentiated constructs and 2D monolayer cultures treated with PD98059 or U0126 showed decreased mineralization, ALP activity, and the expression of the aforementioned genes *BGLAP*, *SPP1*, *COL1A1*, *MAPK1*, *AKT1*, *RUNX2*, and *GLI1* relative to untreated. Differences in ALP activity and osteogenesis related gene expression relative to untreated cells cultured in a 2D monolayer were greater in 3D constructs compared to 2D monolayer cultures. These findings suggest that our bioprinted bone model system offers a more sensitive, biologically relevant drug screening platform than traditional 2D monolayer in vitro testing platforms.

## 1. Introduction

Metabolic bone disease encompasses several disorders that result in abnormalities of bone [1]. These disorders are currently estimated to affect over 200 million people worldwide [2]. Osteoporosis, the loss of bone density resulting from abnormal bone remodeling [3], is the most common metabolic bone disorder [4]. On the opposite end of the spectrum, osteopetrosis is a rare disorder characterized by increased bone density due to a defect in osteoclast resorption [5]. Understanding its pathogenesis can provide deeper insights into the molecular pathways involved in other bone metabolic pathologies, including osteoporosis [6].

In vitro models of bone have become important tools in the development and testing of potential treatments and therapeutic strategies for bone metabolic pathologies [7,8]. For such applications, drugs that either promote [9] or impair [10] bone formation are in high demand. Numerous small molecule drugs capable of regulating osteogenesis through various signaling pathways such as Wnt/β-catenin, Hedgehog, and MEK/ERK mitogen activated kinase (MAPK) have been discovered [11,12,13,14]. Purmorphamine, a small molecule purine derivative and Smoothened (Smo) receptor agonist [15], upregulates GlI1 and Wnt/β-catenin to promote osteogenesis in mesenchymal stem cells (MSCs) by way of the Hedgehog (Hh) signaling pathway [10,11]. Due to its bone regenerative properties, Purmorphamine is being utilized in the development of therapeutic strategies for improving bone repair [16,17]. Purmorphamine has been shown to increase osteogenesis at levels comparable to that of BMP4 administration [18]. Icariin, a natural flavonoid glycoside isolated from *Herba Epimedii*, also promotes osteogenesis by activating the PI3K-AKT and Wnt/β-catenin signaling pathways [19,20]. Icariin has been considered as a potential alternative therapy for bone repair due to its anti-osteoporotic effects [21] and its inhibitory effects on osteoclast differentiation [22]. Three-dimensional scaffolds incorporated with Icariin have been shown to promote early bone formation, as well as exhibit both osteoinductive and osteoconductive properties [21,23]. For MSCs cultured in vitro, Purmorphamine and Icariin treatment at concentrations of 2 μM [24,25] and 1 µM [26,27], respectively, have both been shown to increase ALP activity, calcium deposition, and the expression of *RUNX2*, the transcriptional regulator of osteogenesis and bone matrix protein genes such as osteocalcin (*BGLAP*) and osteopontin (*SPP1*) [24,25,26,27].

MAPK/ERK kinase (MEK)1/2 inhibitors such as PD98059 and U0126 are known to be effective at blocking osteogenesis in MSCs by inhibiting MEK/ERK mitogen activated protein kinase (MAPK) pathways essential during skeletal development and homeostasis, which involve bone formation by osteoblasts and resorption by osteoclasts [28,29]. PD98059 and U0126 have also been shown to increase osteoclastogenesis [28]. For MSCs cultured in vitro, PD98059 treatment at a concentration of 20 μM [30,31] and U0126 treatment at a concentration of 25 µM [32] have both been shown to decrease ALP activity, calcium deposition, and the expression of bone matrix protein genes such as type I collagen (*COL1A1*), bone sialoprotein (*BSP*), *SPP1*, and osteonectin (*SPARC*) [30,31,32].

2D monolayer cultures and preclinical animal models have traditionally been utilized to evaluate the mechanisms of human disease and drug screening. However, traditional 2D cultures provide limited recapitulation of the complex human tissue microenvironment, and animal models often lack clinical translatability to human disease since the efficacy and toxicity of drugs in animal studies do not always predict that of human patients [33]. The increasing significance of 3D bioprinted in vitro models is helping to bridge the gap between 2D cell culture and in vivo animal models [33]. 3D bioprinting has emerged as a valuable tool for producing reliable high throughput models of biological activity for drug discovery [34].

As suggested in our previous study [35], our scaffold-free 3D bioprinted in vitro bone model system can potentially be used for studying repairs of osteochondral defects and drug discovery and response. Similarly produced in vitro bone model systems have also evaluated the osteogenic differentiation capacity [36]; however, few studies have examined osteogenic differentiation capacity in comparison to 2D monolayer culture conditions. Furthermore, the potential use of these bone model systems as an in vitro drug screening platform has yet to be evaluated. In this study, we aimed to establish a framework for the development of an in vitro drug screening platform using 3D bioprinted BM-MSCs undergoing osteogenic differentiation to mimic bone. We investigated the effects of administrating drugs known to promote (Purmorphamine and Icariin) or impair (PD98059 and U0126) osteogenic differentiation in both 3D and 2D culture conditions.

## 2. Results

### 2.1. Osteogenic Differentiation Capacity of 3D Bioprinted Constructs and 2D Monolayer Cultures

**Figure 1:**
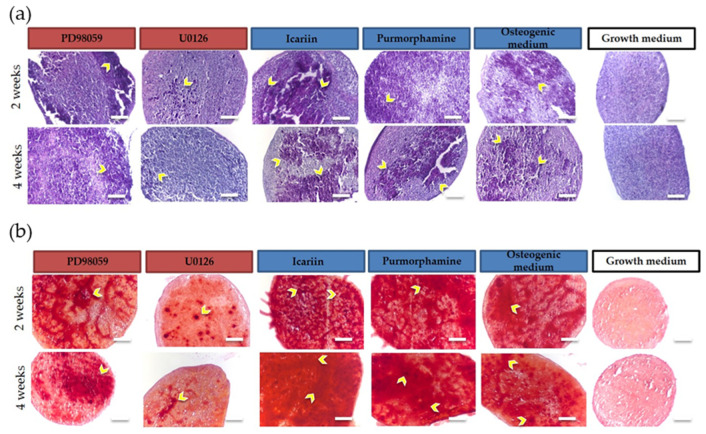
Images of (**a**) H&E and (**b**) Alizarin Red staining of 3D bioprinted constructs (n = 3 per medium condition) at two weeks and four weeks in culture. Cells showed non-uniform calcium deposition, displaying zones of accumulation (dark purple/red; indicated by yellow arrows) indicative of osteogenic differentiation (10× magnification, scale bar = 500 µm).

**Figure 2:**
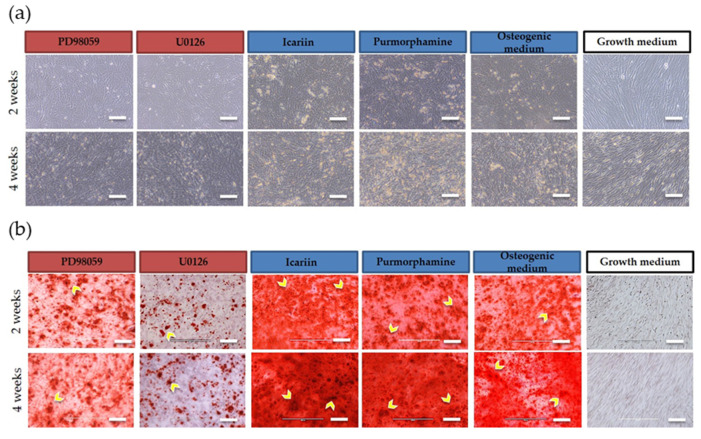
Images before (**a**) and after (**b**) Alizarin Red staining of 2D monolayer cultures (n = 3 per medium condition) at two weeks and four weeks in culture. Cells showed non-uniform calcium deposition, displaying zones of accumulation (dark red; indicated by yellow arrows) indicative of osteogenic differentiation (10× magnification, scale bar = 500 µm).

Mineralization of 3D bioprinted constructs (Figure 1) and 2D monolayer cultures (Figure 2) was exhibited following induction with osteogenic medium as indicated by H&E staining of 3D constructs (Figure 1a) and brightfield imaging of 2D monolayer cultures (Figure 2a). Positive Alizarin Red staining of 3D constructs (Figure 1b) and 2D monolayer cultures (Figure 2b) was also exhibited.

In both 3D and 2D culture conditions, calcium deposition was non-uniform, displaying zones of accumulated deposition. The intensity of calcified areas appeared to increase in the Purmorphamine or Icariin treated 3D constructs and 2D monolayer cultures in comparison to the osteogenic medium controls at both Weeks 2 and 4. Alizarin Red staining appeared to decrease in intensity in the PD98059 or U0126 treated 3D constructs and 2D monolayer cultures in comparison to the osteogenic medium controls at both Weeks 2 and 4. No mineralization was observed in cells cultured in BM-MSC growth medium as indicated by negative Alizarin Red staining. Overall, both 3D bioprinted constructs and 2D monolayer cultures were responsive to drug treatment and maintained their osteogenic potential as demonstrated by their capability to mineralize the extracellular matrix at Weeks 2 and 4.

### 2.2. Differential Alkaline Phosphatase Activity of 3D Bioprinted Constructs and 2D Monolayer Cultures

**Figure 3:**
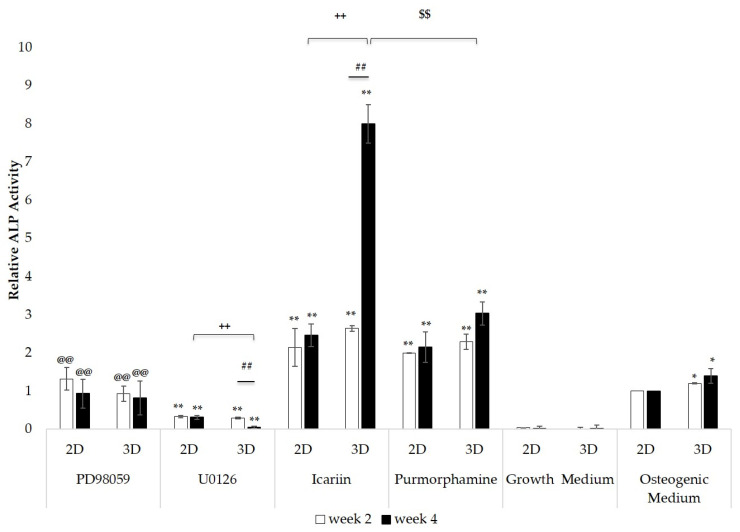
ALP activity was measured in bioprinted constructs (3D) and in monolayer cultures (2D) after two weeks and four weeks in culture. The graph shows fold changes in ALP activity relative to the osteogenic medium control grown in 2D culture conditions. Indicated error bars represent the standard deviation, and significance was determined by one-way ANOVA with Tukey’s multiple comparison test. * *p* < 0.05 compared to the osteogenic medium control in 2D culture conditions, ** *p* < 0.01 compared to the osteogenic medium control in 2D culture conditions, ## *p* < 0.01 between 3D bioprinted constructs at Week 2 and Week 4, ++ *p* < 0.01 between 3D bioprinted constructs and 2D monolayer cultures at Week 4, $$ *p* < 0.01 between Icariin and Purmorphamine treated 3D bioprinted constructs at Week 4, @@ *p* < 0.01 compared to U0126 treated 3D bioprinted constructs and 2D monolayer cultures at Week 2 and Week 4. n = 3 for each treatment.

ALP activity of 3D bioprinted constructs and 2D monolayer cultures at two and four weeks is presented in Figure 3 as the fold change relative to the osteogenic medium control cultured in 2D culture conditions. 3D constructs and 2D monolayer cultures in BM-MSC growth medium showed significantly lower ALP activity than those cultured in osteogenic medium (** *p* < 0.01). ALP activity of 3D constructs cultured in the osteogenic medium control was significantly greater than that of the 2D monolayer culture grown in the osteogenic medium control (* *p* < 0.05). Icariin or Purmorphamine treated 3D constructs and 2D monolayer cultures showed a significant increase in ALP activity (** *p* < 0.01). U0126 treated 3D constructs and 2D monolayer cultures showed a significant decrease in ALP activity (** *p* < 0.01). No significant changes in ALP activity were observed in PD98059 treated 3D constructs and 2D monolayer culture. ALP activity of Icariin or Purmorphamine treated 3D constructs and 2D monolayer cultures was significantly greater than those treated with PD98059 or U0126 (** *p* < 0.01). No significant changes in ALP activity were observed in 2D monolayer cultures and Purmorphamine or PD98059 treated 3D constructs between Week 2 and Week 4. However, Icariin treated 3D constructs showed a significant increase in ALP activity between Week 2 and Week 4, and U0126 treated 3D construct cultures showed a significant decrease in ALP activity between Week 2 and Week 4 (** *p* < 0.01). At Week 4, ALP activity of Icariin treated 3D constructs was significantly greater than that of Icariin treated 2D monolayer cultures and Purmorphamine treated 3D constructs (** *p* < 0.01). ALP activity of U0126 treated 3D constructs was significantly lower than that of U0126 treated 2D monolayer cultures at Week 4 (** *p* < 0.01). At both Week 2 and Week 4, ALP activity of U0126 treated 3D constructs and 2D monolayer cultures was significantly lower than PD98059 treated 3D constructs and 2D monolayer cultures (** *p* < 0.01). Overall, both 3D and 2D culture conditions were responsive to drug treatment; however, differences in ALP activity relative to the osteogenic medium control grown in 2D culture conditions were greater in 3D bioprinted constructs, suggesting that 3D culture conditions provide a more sensitive response to drug treatment.

### 2.3. Differential Gene Expression of 3D Bioprinted Constructs and 2D Monolayer Cultures

**Figure 4:**
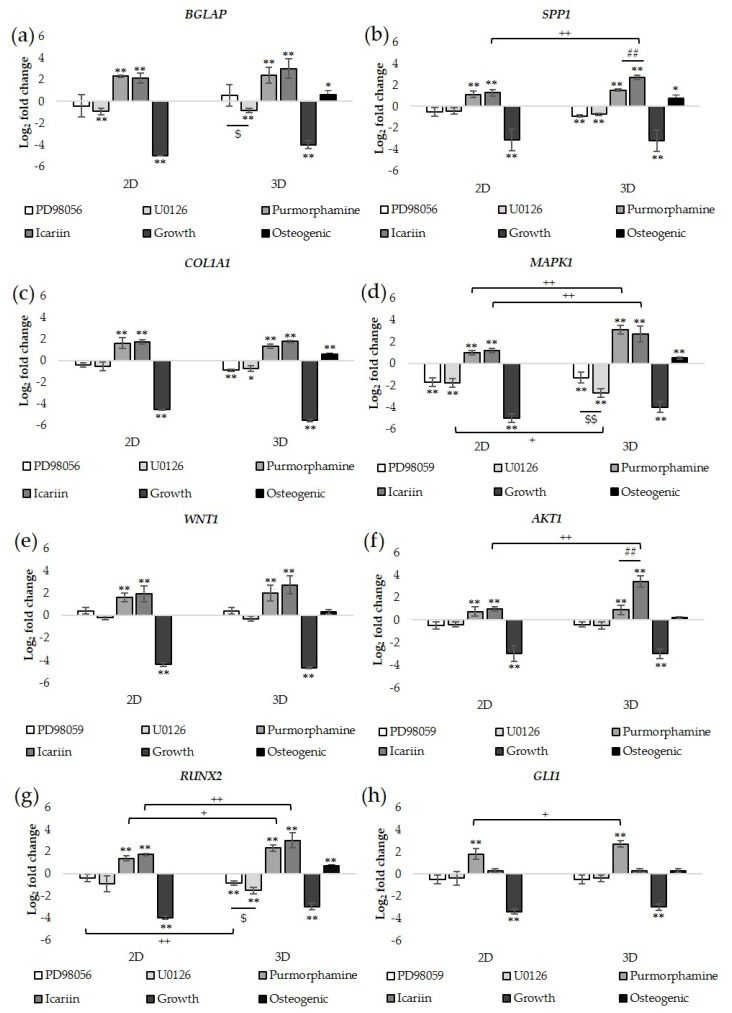
For treated and untreated bioprinted constructs (3D) and monolayer cultures (2D), RT-qPCR was used to determine the expression of osteogenesis associated genes (**a**) *BGLAP*, (**b**) *SPP1*, (**c**) *COL1A1*, (**d**) *MAPK1*, (**e**) *WNT1*, (**f**) *AKT1*, (**g**) *RUNX2*, and (**h**) *GLI1* after four weeks of culture. The graph shows the log_2_ fold changes in gene expression relative to the osteogenic medium control grown in 2D culture conditions. Indicated error bars represent the standard deviation, and significance was determined by one-way ANOVA with Tukey’s multiple comparison test. * *p* < 0.05 compared to osteogenic medium control in 2D culture conditions, ** *p* < 0.01 compared to osteogenic medium control in 2D culture conditions, + *p* < 0.05 between 3D constructs and 2D monolayer cultures, ++ *p* < 0.01 between 3D constructs and 2D monolayer cultures, ## *p* < 0.01 between Icariin and Purmorphamine 3D constructs, $ *p* < 0.05 between U0126 and PD98059 treated 3D constructs, and $$ *p* < 0.01 between U0126 and PD98059 treated 3D constructs. n = 3 for each treatment.

The gene expression of bone formation markers (*BGLAP*, *SPP1*, and *COL1A1*), signaling molecules (*MAPK1*, *WNT1*, and *AKT1*), and transcription factors (*RUNX2* and *GLI1*) that regulate osteogenic differentiation at Week 4 is presented as the log_2_ fold change relative to the osteogenic medium control grown in 2D culture conditions (Figure 4a–h).

For 3D bioprinted constructs and 2D monolayer cultures treated with BM-MSC growth medium, a 3–6-fold decrease in the expression of all genes evaluated was observed relative to those cultured in osteogenic medium (** *p* < 0.01). The expression of the bone formation markers *BGLAP*, *SPP1*, and *COL1A1* was significantly greater in Icariin or Purmorphamine treated 3D constructs and 2D monolayer cultures compared to those treated with U0126 or PD98059 (** *p* < 0.01; Figure 4a–c). *BGLAP*, *SPP1*, and *COL1A1* expression increased significantly in 3D constructs treated with Icariin (3.0 ± 0.9, 2.7 ± 0.2, 1.8 ± 0.1) or Purmorphamine (2.4 ± 0.7, 1.5 ± 0.1, 1.3 ± 0.2) and 2D monolayer cultures treated with Icariin (2.2 ± 0.5, 1.3 ± 0.3, 1.7 ± 0.2) or Purmorphamine (2.3 ± 0.1, 1.1 ± 0.3, 1.6 ± 0.5) (** *p* < 0.01). *SPP1* expression in Icariin treated 3D constructs was significantly greater than that of Purmorphamine treated 3D constructs or Icariin treated 2D monolayer cultures (** *p* < 0.01). No significant changes in *BGLAP* and *COL1A1* expression were observed between Purmorphamine and Icariin treated 3D constructs or 2D monolayer cultures.

3D constructs treated with U0126 showed a decrease in the expression of *BGLAP*, *SPP1*, and *COL1A1* (−0.9 ± 0.2, −0.7 ± 0.1, −0.8 ± 0.3). For 3D constructs treated with PD98059, the expression of *SPP1* and *COL1A1* decreased (−0.9 ± 0.1, −0.9 ± 0.1). *BGLAP* expression in U0126 treated 3D constructs was significantly lower than that of PD98059 treated 3D constructs, which showed an increase in *BGLAP* expression (0.5 ± 0.2) (* *p* < 0.05). The expression of *BGLAP*, *SPP1*, and *COL1A1* decreased in 2D monolayer cultures treated with U0126 (−0.9 ± 0.3, −0.4 ± 0.1, −0.5 ± 0.4) or PD98059 (−0.4 ± 0.3, −0.5 ± 0.4, −0.4 ± 0.1). No significant changes in *BGLAP*, *SPP1*, and *COL1A1* expression were observed between U0126 and PD98059 treated 3D constructs or 2D monolayer cultures.

The expression of the signaling molecules *MAPK1*, *WNT1*, and *AKT1* was also significantly greater in Icariin and Purmorphamine treated 3D constructs and 2D monolayer cultures compared to those treated with U0126 or PD98059 (** *p* < 0.01; Figure 4d–f). *MAPK1*, *WNT1*, and *AKT1* expression increased significantly in 3D constructs treated with Icariin (2.7 ± 0.7, 2.7 ± 0.8, 3.4 ± 0.5) or Purmorphamine (3.1 ± 0.4, 2.0 ± 0.9, 0.9 ± 0.4) and 2D monolayer cultures treated with Icariin (1.2 ± 0.2, 1.9 ± 0.7, 1.0 ± 0.2) or Purmorphamine (1.0 ± 0.2, 1.6 ± 0.4, 0.8 ± 0.4). *MAPK1* expression in Icariin or Purmorphamine treated 3D constructs was significantly greater than that of Icariin or Purmorphamine treated 2D monolayer cultures (** *p* < 0.01). *AKT1* expression in Icariin treated 3D constructs was significantly greater than that of the Icariin treated monolayer culture and Purmorphamine treated 3D constructs and 2D monolayer cultures (** *p* < 0.01). No significant changes in *WNT1* expression were observed between Purmorphamine and Icariin treated 3D constructs or 2D monolayer cultures.

*MAPK1*, *WNT1*, and *AKT1* expression was decreased in U0126 treated 3D constructs (−2.7 ± 0.4, −0.3 ± 0.2, −0.5 ± 0.3) and 2D monolayer cultures (−1.8 ± 0.4, −0.2 ± 0.2, −0.4 ± 0.2). *MAPK1* and *AKT1* expression was decreased in PD98059 treated 3D constructs (−1.3 ± 0.5, -0.3 ± 0.2) and 2D monolayer cultures (−1.7 ± 0.2, −0.4 ± 0.1); however, *WNT1* expression increased (0.4 ± 0.3, 0.4 ± 0.3). *MAPK1* expression in U0126 treated 3D constructs was significantly lower than that of PD98059 treated 3D constructs (* *p* < 0.05) or U0126 treated 2D monolayer cultures (** *p* < 0.01). No significant changes in *WNT1* and *AKT1* expression were observed between U0126 and PD98059 treated 3D constructs or 2D monolayer cultures.

The expression of the transcription factors *RUNX2* and *GLI1* was significantly greater in Purmorphamine treated 3D constructs and 2D monolayer cultures compared to those treated with U0126 or PD98059 (** *p* < 0.01; Figure 4g,h). *RUNX2* and *GLI1* expression increased in 3D constructs treated with Icariin (3.0 ± 0.7, 0.3 ± 0.8) or Purmorphamine (2.3 ± 0.3, 2.7 ± 0.3) and 2D monolayer cultures treated with Icariin (1.7 ± 0.1, 0.3 ± 0.7) or Purmorphamine (1.4 ± 0.2, 1.8 ± 0.5). *RUNX2* expression in Icariin or Purmorphamine treated 3D constructs was significantly greater than that of Icariin or Purmorphamine treated 2D monolayer cultures (* *p* < 0.05 for Purmorphamine treatment, ** *p* < 0.01 for Icariin treatment). *GLI1* expression in Purmorphamine treated 3D constructs was significantly greater than that of Purmorphamine treated 2D monolayer cultures (* *p* < 0.05).

*RUNX2* and *GLI1* expression was decreased in 2D monolayer cultures treated with U0126 (−0.9 ± 0.7, −0.4 ± 0.6) or PD98059 (−0.4 ± 0.3, −0.5 ± 0.4). *RUNX2* expression in U0126 treated 3D constructs (−1.5± 0.2) was significantly lower than that of PD98059 treated 3D constructs (−0.9± 0.7) (* *p* < 0.05). *RUNX2* expression in PD98059 treated 3D constructs was significantly greater than PD98059 treated 2D monolayer cultures (** *p* < 0.01). No significant differences in *GLI1* expression were observed in Icariin treated 3D constructs and 2D monolayer cultures compared to those treated with U0126 or PD98059. The expression of *SPP1*, *BGLAP*, *COL1A1*, *MAPK1*, and *RUNX2* increased 3D constructs cultured in osteogenic medium control showed higher expression compared to 2D monolayer cultures cultured in the osteogenic medium control (* *p* < 0.05 for *SPP1* and *BGLAP*; ** *p* < 0.01 for *COL1A1*, *MAPK1*, and *RUNX2*). Overall, both 3D and 2D culture conditions were responsive to drug treatment; however, differences in osteogenesis related gene expression following drug treatment relative to the osteogenic medium control grown in 2D culture conditions were greater in 3D bioprinted constructs, suggesting that 3D culture conditions provide a more sensitive response to drug treatment.

## 3. Discussion

**Figure 5:**
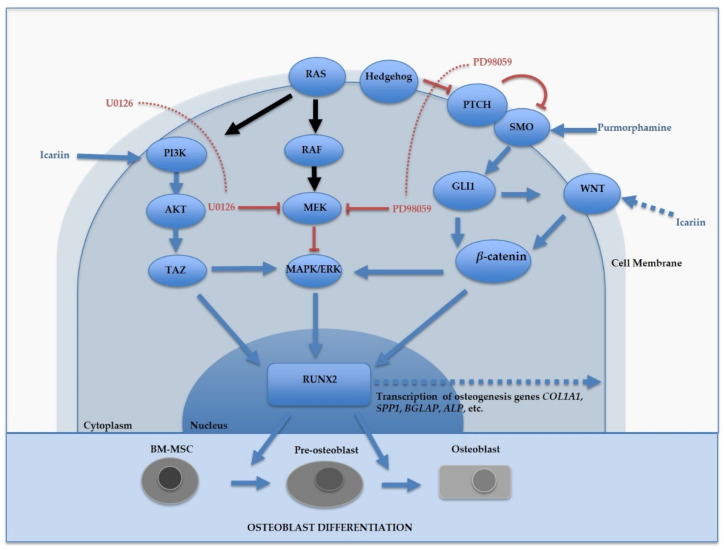
The effect of Icariin, Purmorphamine, U0126, and PD98059 on osteogenic signaling pathways. Icariin activates PI3K/Akt, promoting downstream *RUNX2* expression and osteogenic lineage commitment. Purmorphamine stimulates Smoothened (Smo), activating the Hedgehog (Hh) signaling pathway, resulting in the upregulation of its downstream target gene *GLI1*, as well as the Wnt/β-catenin signaling pathway, leading to bone formation and the promotion of osteogenic lineage commitment. Icariin also regulates osteogenic commitment via the Wnt/β-catenin pathway. U0126 and PD98059 act as MAPK/ERK mitogen activated kinase (MAPK) inhibitors, resulting in inhibition of osteogenic lineage commitment.

This study demonstrates the utility of our scaffold-free 3D bioprinted bone model systems as an in vitro tool to screen multiple drugs that promote or impair osteogenic differentiation. Small molecule drugs, such as the ones utilized in this study, can help in providing a better understanding about the molecular mechanisms involved in osteogenesis [9,10]. Figure 5 shows a diagram highlighting the effects of Icariin, Purmorphamine, U0126, and PD98059 on various signaling pathways responsible for regulating osteogenesis.

The results of this study suggest that Purmorphamine and Icariin have an enhancing effect on osteogenesis, as shown by increased mineralization (Alizarin Red), an increase in ALP activity, and osteogenesis related gene expression. Purmorphamine promotes osteogenic lineage commitment by activating the Hedgehog or Wnt/β-catenin signaling pathways [10,11], and Icariin promotes osteogenic lineage commitment by activating the PI3K/Akt signaling pathway and regulating Wnt signaling [19,20].

This correlates with our gene expression analysis, which showed the highest level of *AKT1* expression in Icariin treated 3D constructs and 2D monolayer cultures. The expression of *GLI1*, a downstream target gene of the Hedgehog pathway, was greatest in Purmorphamine treated 3D constructs and 2D monolayer cultures. For both 3D constructs and 2D monolayer cultures treated with Icariin or Purmorphamine, there was an increase in the expression of *WNT1*. Both PI3K/Akt and Wnt/β-catenin signaling pathways result in the downstream activation of the Runt-related transcription factor (*RUNX2*) that is required for the expression of multiple osteogenic genes such as *COL1A1*, the main collagen expressed by osteoblasts [37], the mature osteoblast markers, *SPP1* and *BGLAP* [38], and *ALP*, the major enzyme involved in osteoblast mineralization [39]. In addition to promoting osteogenic differentiation of MSCs into osteoblasts, Icariin and Purmorphamine inhibit bone resorption by suppressing osteoclastogenesis and the bioactivity of the osteoclasts [40,41]. For diseases such as osteoporosis resulting from excessive bone resorption by osteoclasts, drugs such as Purmorphamine and Icariin could be utilized in potential treatments [42,43]. Although both drugs Icariin and Purmorphamine promote osteogenesis, the results of this study suggest that Icariin is more effective. At Week 4, ALP activity, as well as the expression of *SPP1* and *AKT1* were significantly greater in Icariin treated 3D constructs compared to Purmorphamine treated 3D constructs. However, differences in ALP activity and gene expression between Icariin and Purmorphamine treated 2D monolayer cultures were marginal. These findings suggest that 3D bioprinted constructs are more sensitive to drug treatment than 2D monolayer cultures.

Treatment with U0126 or PD98059 had an inhibitory effect on osteogenesis as shown by decreased mineralization, ALP activity, and the expression of osteogenesis related genes. Both drugs PD98059 and U0126 are known to be effective in blocking osteogenesis by inhibiting the MEK/ERK MAPK pathway that is critical for the activation of *RUNX2*, the master regulator of osteogenesis [28,29]. This correlates with our gene expression analysis, which showed decreased expression of *MAPK*, as well as *RUNX2* expression in both 3D constructs and 2D monolayer culture treated with U0126 and PD98059. In addition to impairing osteogenic differentiation of MSCs into osteoblasts, PD98059 and U0126 have been shown to promote osteoclast differentiation [44]. As a result, these drugs could be utilized in potential treatments for diseases resulting from defective osteoblast differentiation and bone resorption such as osteopetrosis [44]. As previous studies have shown [45,46,47], the results of this study also suggest that U0126 is more effective at inhibiting *MAPK* and impairing osteogenesis than PD98059. At both Weeks 2 and 4, ALP activity of U0126 treated 3D constructs and 2D monolayer cultures was lower than PD98059 treated 3D constructs and 2D monolayer cultures. At Week 4, the expression of *BGLAP* and *MAPK* was significantly lower in U0126 treated 3D bioprinted constructs compared to PD98059 treated 3D constructs; however, there were only marginal differences in the expression of *BGLAP* and *MAPK* between U0126 and PD98059 treated 2D monolayer cultures.

3D based cell systems show differences in drug sensitivity as a result of differential gene and/or protein expression in comparison to 2D monolayer cultures [48]. 3D based cell systems have become an integral part of drug discovery/screening platform development since they better mimic the in vivo microenvironment [49]. In this study, differences in ALP activity and osteogenesis related gene expression relative to the osteogenic medium control grown in 2D culture conditions were greater in 3D constructs compared to 2D monolayer cultures. The expression of *SPP1*, *RUNX2*, *MAPK1*, and *AKT1* was significantly greater in Icariin treated 3D constructs compared to Icariin treated 2D monolayer cultures. *MAPK1* expression was significantly lower in U0126 treated 3D constructs compared to U0126 treated 2D monolayer cultures. In conclusion, the results of this study suggest that our 3D bioprinted bone model system offers a more sensitive, biologically relevant drug screening platform than traditional 2D monolayer in vitro testing platforms. Additional design considerations can be incorporated into future iterations, such as the addition of vasculature to more faithfully recapitulate the physiopathology of bone [50]; however, the first efforts of utilizing these bioprinted bone model systems shown in this study help to advance and improve the growing area of scaffold-free bioprinting, which could one day become a go-to tool for drug screening and tissue engineering.

## 4. Materials and Methods

### 4.1. Materials and Reagents

Stock solutions of PD98059 (20 mM), U0126 (25 mM), Icariin (10 mM), and Purmorphamine (2 mM) (Sigma Aldrich, St. Louis, MO, USA) were all prepared in anhydrous DMSO (Sigma Aldrich) the day of the experiment. The prepared aliquots were stored at −20 °C until use.

### 4.2. Cell Culture

#### 4.2.1. Spheroid Formation

BM-MSC expansion and spheroid formation have been previously described in detail [28]. In brief, cryopreserved-thawed BM-MSCs at passage 4 (LifeNet Health, Virginia Beach, VA, USA) were cultured in BM-MSC growth medium supplemented with fetal bovine serum, rh FGF, rh IGF-1, and l-alanyl-l-glutamine (ATCC, Manassas, VA, USA) to reach 70% confluence. Cultured cells were then harvested and seeded into 96 well round bottom low adhesion plates (Sumitomo Bakelite, Tokyo, Japan) at 2.5 × 10^4^ cells/well. Constructs were designed using the Bio 3D Designer software provided with the scaffold-free 3D bioprinter, Regenova^®^ (Cyfuse Biomedical KK, Tokyo, Japan), prior to bioprinting. For each produced construct, a total of 27 formed spheroids (500 μm in diameter) were bioprinted in three layers (9 spheroids per layer) following a 3 × 3 × 3 cuboidal configuration. Once spheroids were adequately fused, the resulting constructs were removed from the needle array and transferred to individual wells of low adhesion 24 well plates (Corning, Corning, NY, USA).

#### 4.2.2. Cell Seeding on the Monolayer

At passage 4, trypsin/EDTA (0.05%/0.02%, ATCC) dissociated BM-MSCs were seeded at a seeding density of 5 × 10^3^ cells/cm^2^ onto flat bottom 24 well tissue culture treated plates (Corning). Cells were grown to 70% confluency in BM-MSC growth medium (ATCC).

### 4.3. Osteogenic Drug Screening Platform

**Figure 6:**
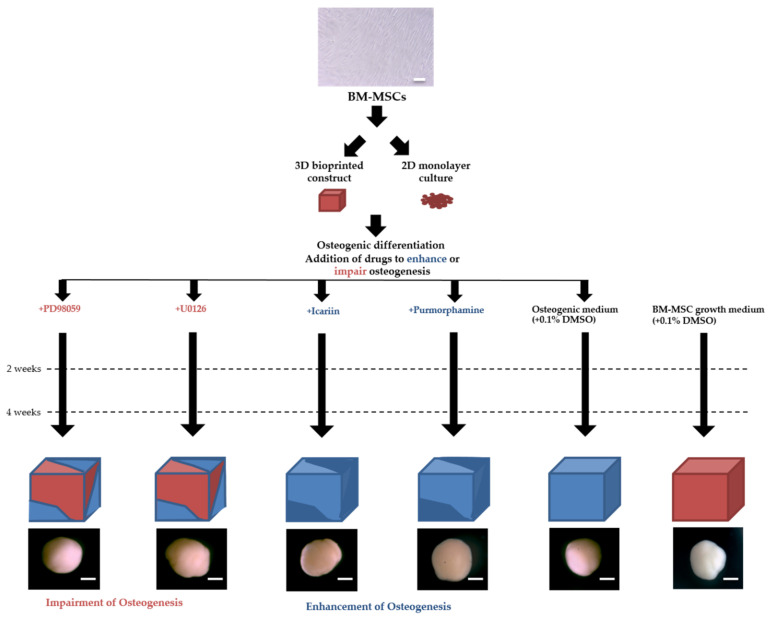
BM-MSC derived 3D bioprinted constructs and 2D monolayer cultures were cultured in BM-MSC osteogenic medium treated with drugs known to promote (Icariin-1 μM, Purmorphamine-2 μM) or impair (PD98059–20 μM, U0126–25 μM) osteogenesis. Vehicle controls of osteogenic medium and BM-MSC growth medium treated with 0.1% DMSO were also prepared. Osteogenesis was evaluated in each culture condition after 2 weeks and 4 weeks in culture. A representative image of bioprinted constructs in each culture condition is shown at 4.5× magnification (scale bar = 500 μm).

Figure 6 illustrates our osteogenic drug screening platform. Working solutions of PD98059 (20 μM), U0126 (25 μM), Icariin (1 μM), and Purmorphamine (2 μM) were prepared from stock aliquots in hMSC osteogenic medium composed of osteogenic basal medium supplemented with dexamethasone, l-glutamine, ascorbate, penicillin/streptomycin, mesenchymal cell growth supplement, and β-glycerophosphate (Lonza). Vehicle controls of osteogenic medium and BM-MSC growth medium (ATCC) treated with 0.1% DMSO were also prepared. 3D bioprinted constructs and 2D monolayer cultures were incubated in each culture condition for 4 weeks. The medium was replaced every 3 days. Working solutions were prepared from frozen stock aliquots immediately before use. Bioprinted constructs were imaged in each culture condition at 4.5× magnification using an SZ61 Microscope (Olympus, Tokyo, Japan).

### 4.4. Alkaline Phosphatase Activity

At 2 and 4 weeks, 3D bioprinted constructs and 2D monolayer cultures (n = 3 for each culture condition) were harvested and resuspended in ALP Assay buffer (Abcam, Cambridge, MA, USA). For further dissociation, bioprinted constructs were homogenized with CK28 hard tissue homogenizing beads by applying two 30 s pulses at 5000 rpm using a Minilys Homogenizer (Precellys-Bertin Technologies, Rockville, MD, USA). Lysates were placed into ice for 1 min in between pulses to prevent overheating. Following homogenization, samples were then centrifuged at 14,000 rpm for 15 min at 4 °C. The resulting supernatants were collected and used for quantification of ALP activity using an Alkaline Phosphatase Assay Kit (Abcam) according to the manufacturer’s protocol. Optical density was measured at 405 nm using a Multiskan GO Microplate Spectrophotometer (Thermo Fisher Scientific, Rockford, IL, USA). Data are presented as the fold change of ALP activity relative to the osteogenic medium control grown in 2D culture conditions.

### 4.5. Histological Analysis

At 2 and 4 weeks, 2D monolayer cultures (n = 3 for each treatment condition) were rinsed twice in DPBS and fixed using 4% paraformaldehyde solution (Sigma Aldrich) for 20 min. The wells were subsequently washed 3 times in deionized water and stained with 2% Alizarin Red S (ScienCell, Carlsbad, CA, USA) for 20 min at room temperature to assess mineralization. Wells were washed 3 additional times in deionized water to remove residual Alizarin Red. Representative wells were imaged before and after Alizarin Red staining using a BX41 Microscope (Olympus) at 10× magnification.

At 2 and 4 weeks, bioprinted constructs (n = 3 for each treatment condition) were fixed in 10% neutral buffered formalin (Cardinal Health, Virginia Beach VA, USA) for 24 h, dehydrated with graded ethanol washes, cleared with Citrisolv (Thermo Fisher Scientific), and embedded in paraffin (Thermo Fisher Scientific). Constructs were sectioned longitudinally at a thickness of 7 μm using an RM 2135 Leica microtome (Leica Microsystems Inc., Columbia, MD, USA) and fixed onto positively charged slides (VWR, West Chester, PA, USA). Following deparaffinization and rehydration with Citrisolv and ethanol, mineralization was assessed with Hematoxylin and Eosin Y (H&E; Thermo Fisher Scientific) and 2% Alizarin Red S (ScienCell). Slides were coverslipped with Permount (Thermo Fisher Scientific) and imaged using a BX41 Microscope at 10× magnification.

### 4.6. RNA isolation and RT-qPCR

At 4 weeks, 2D monolayer cultures and bioprinted constructs were lysed in RLT buffer (Qiagen, Valencia, CA, USA). For bioprinted constructs, the resulting lysate was homogenized with CK28 hard tissue homogenizing beads (see above). Total RNA was extracted using the RNeasy mini kit (Qiagen) according to the manufacturer’s instructions. RNA (500 ng) was reverse transcribed to cDNA using the and PrimeScript RT reagent kit (Takara Bio, Shiga, Japan) and a Veriti Thermocycler (Applied Biosystems, Foster City, CA, USA) according to the manufacturer’s instructions. Quantitative reverse transcription PCR (RT-qPCR) was performed using synthesized cDNA and the QuantiNova SYBR Green RT-PCR Kit (Qiagen) on a StepOnePlus Real Time PCR System (Applied Biosystems) according to the manufacturer’s instructions. The custom primer sequences used for qRT-PCR are listed in Table 1. Data were normalized to GAPDH and are presented as the log_2_ fold change relative to the osteogenic medium control grown in 2D culture conditions calculated using the *2*^−*ΔΔCt*^ method with Step One software (Version 2.3, Applied Biosystems).

### 4.7. Statistical Analysis

Three biological replicates of each culture condition were used to determine statistical significance. Error bars represent the standard deviation. The analyses were performed with SPSS 24.0 statistical software (SPSS, Chicago, IL, USA). A one-way ANOVA with Tukey’s multiple comparison test was used to determine statistical significance with 95% confidence and *p* < 0.05 for statistical significance.

## Figures and Tables

**Table 1:** List of primer sequences.

Gene	Primer Sequences
*BGLAP*	Forward: 5′-CACTCCTCGCCCTATTGGC-3′
	Reverse: 5′-CCCTCCTGCTTGGACACAAAG-3′
*SPP1*	Forward: 5′-GAAGTTTCGCAGACCTGACAT-3′
	Reverse: 5′-GTATGCACCATTCAACTCCTCG-3′
*COL1A1*	Forward: 5′-CAGCCGCTTCACCTACAGC-3′
	Reverse: 5′-TTTTGTATTCAATCACTGTCTTGCC -3′
*MAPK1*	Forward: 5′-ACACCAACCTCTCGTACATCGG-3′
	Reverse: 5′-TGGCAGTAGGTCTGGTGCTCAA-3′
*WNT1*	Forward: 5′-CTCTTCGGCAAGATCGTCAACC-3′
	Reverse: 5′-CGATGGAACCTTCTGAGCAGGA-3′
*AKT1*	Forward: 5′-TGGACTACCTGCACTCGGAGAA-3′
	Reverse: 5′-GTGCCGCAAAAGGTCTTCATGG-3′
*RUNX2*	Forward: 5′-TCAACGATCTGAGATTTGTGGG-3′
	Reverse: 5′-GGGGAGGATTTGTGAAGACGG-3′
*GLI1*	Forward: 5′-AGCCTTCAGCAATGCCAGTGAC-3′
	Reverse: 5′-GTCAGGACCATGCACTGTCTTG-3′
*GAPDH*	Forward: 5′-ACCACAGTCCATGCCATCAC-3′
	Reverse: 5′-TCCACCACCCTGTTGCTGTA-3′

*BGLAP*, osteocalcin; *SPP1*, osteopontin; *COL1A1*, type I collagen; *MAPK1*, mitogen-activated protein kinase 1; *WNT1*, proto-oncogene protein Wnt-1; *AKT1*, RAC-alpha serine/threonine-protein kinase; *RUNX2*, Runt related transcription factor 2; *GLI1*, GLI family zinc finger 1; *GAPDH*, glyceraldehyde 3-phosphate dehydrogenase.
